# Anti-tumor activity of a recombinant measles virus against canine lung cancer cells

**DOI:** 10.1038/s41598-023-42305-9

**Published:** 2023-10-24

**Authors:** Kei Tamura, Tomoko Fujiyuki, Kanako Moritoh, Hayato Akimoto, Keigo Iizuka, Hiroki Sato, Kazushi Asano, Misako Yoneda, Chieko Kai

**Affiliations:** 1 Laboratory Animal Research Center, The Institute of Medical Science, The University of Tokyo, 4-6-1, Shirokanedai, Minato-Ku, Tokyo, 108-8639 Japan; 2 Laboratory of Veterinary Surgery, Department of Veterinary Medicine, College of Bioresource Sciences, Nihon University, 1866 Kameino, Fujisawa, Kanagawa 252-0880 Japan; 3 Present Address: Institute of Industrial Science, The University of Tokyo, 4-6-1, Komaba, Meguro-ku, Tokyo, 153-8505 Japan; 4 Institute of Industrial Science, The University of Tokyo, Tokyo, Japan

**Keywords:** Oncology, Cancer, Cancer therapy, Virology, Measles virus

## Abstract

Canine primary lung cancer with metastasis has a poor prognosis with no effective treatment. We previously generated a recombinant measles virus (MV) that lost binding affinity to a principal receptor, SLAM, to eliminate its virulence as a new cancer treatment strategy. The virus, rMV-SLAMblind, targets nectin-4, recently listed as a tumor marker, and exerts antitumor activity against nectin-4-positive canine mammary cancer and urinary bladder transitional cell carcinoma cells. However, the effectivity of rMV-SLAMblind for other types of canine cancers is still unknown. Here we evaluated the antitumor effect of rMV-SLAMblind to canine lung cancer. Nectin-4 is expressed on three canine lung cancer cell lines (CLAC, AZACL1, AZACL2) and rMV-SLAMblind was able to infect these cell lines. CLAC cells showed reduced cell viability after virus infection. In the CLAC xenograft nude mouse model, intratumoral administration of rMV-SLAMblind significantly suppressed tumor growth. In rMV-SLAMblind-treated mice, natural killer cells were activated, and *Cxcl10* and *Il12a* levels were significantly increased in comparison with levels in the control group. In addition, the depletion of NK cells reduced the anti-tumor effect. To understand difference in efficacy among canine lung cancer cell lines, we compared virus growth and gene expression pattern after virus treatment in the three canine lung cancer cell lines; virus growth was highest in CLAC cells compared with the other cell lines and the induction of interferon (IFN)-beta and IFN-stimulated genes was at lower levels in CLAC cells. These results suggested that rMV-SLAMblind exhibits oncolytic effect against some canine lung cancer cells and the cellular response after the virus infection may influence its efficacy.

## Introduction

Lung cancer is a common cancer in humans and has a high mortality rate^1^. Lung cancer is mainly divided into two subtypes, small cell lung carcinoma and non-small cell lung carcinoma, the latter of which includes squamous cell carcinoma, adenocarcinoma and large-cell carcinoma^2^. Treatment options and survival rates for lung cancer depend on the size of the tumor, the presence or absence of metastases and the condition of the patient. The 5-year survival rate of early stage lung cancer patients (stage 1A) treated with surgical resection is 77–92%^2^. As the disease progresses, it is recommended to combine surgical resection with radiation therapy and/or chemotherapy. For patients with metastases to regional lymph nodes and/or distant metastases or multiple tumors (stage 3–4), surgical resection is not recommended^3^. Patients with stage 4 cancer are treated with chemotherapy and molecular target drugs; however, the 5-year survival rate of these patients is 10% or less^2^.

Lung cancer is metastatic also in dogs, and one of important diseases in veterinary medicine. Canine lung cancer has a high metastatic rate; approximately 71% of canine lung cancer cases show evidence of local vascular or lymphatic invasion and 23% have distant metastasis beyond hilar lymph nodes^4,5^. The median survival time (MST) of canine lung cancer cases with no clinical sign is 545 days, whereas the MST with clinical signs such as cough, anorexia and dyspnea is 240 days^6^. The high metastatic rate and poor prognosis of canine lung cancer is because of the lack of specific clinical signs and the low frequency of routine X-rays and blood tests. Surgical resection is recommended for canine lung cancer with no metastases and no lymph node infiltration. However, in advanced cancer cases with metastases or multiple tumors, treatment is limited to chemotherapy such as cyclophosphamide and mitoxantrone but is less effective^7,8^.

Oncolytic virotherapy is a new strategy for cancer treatment. Oncolytic viruses have an advantage in selectively infecting and killing tumor cells. Many oncolytic viruses have been examined in clinical trials and several viruses, such as Telomelysin^®^ (OBP-301), Talimogene laherparepvec (T-VEC) and Reolysin, have been approved^9–11^. We previously generated an oncolytic virus from a measles virus (MV)^12^, which belongs to the genus Morbillivirus in the family Paramyxoviridae, that exhibits oncolytic activity. We genetically modified the hemagglutinin (H) protein of MV, which is required for viral entry into cells, by introducing one amino acid substitution. The mutation on the H protein successfully eliminated virus binding ability to an immune cell receptor, SLAM^13^, and led to attenuated virulence in susceptible monkeys^12^. The recombinant MV, termed rMV-SLAMblind, uses nectin-4 but not SLAM as a receptor^12^. The expression of nectin-4 is restricted to placenta tissues and is not expressed at high levels in healthy individuals^14^. In contrast, nectin-4 is upregulated in various tumors such as thyroid, ovarian, lung, colon, pancreatic, urothelial carcinoma, esophageal, gastric, hepatocellular carcinoma and breast cancers^15–24^. We investigated the oncolytic activity of rMV-SLAMblind in various nectin-4-positive human cancer cell lines including breast cancer, triple negative breast cancer, lung cancer, colon cancer and pancreatic cancer, and showed that rMV-SLAMblind exerted strong cytotoxicity in vitro and an inhibitory effect on tumor growth in xenograft models^12,25–28^.

The amino acid sequence of nectin-4 in the domain critical for binding to MV-H protein is completely conserved between human and dog^29^. We demonstrated that rMV-SLAMblind can infect canine mammary cancer cells and urinary bladder transitional cell carcinoma expressing nectin-4 and exerts antitumor effect in vivo^29,30^. However, the effect of rMV-SLAMblind on canine lung cancer is still unknown. In the present study, we evaluated the potential anti-tumor effect of rMV-SLAMblind against canine lung cancer in vitro and in vivo*.*

## Results

### Antitumor activity of rMV-SLAMblind against canine lung cancer cells

**Figure 1:**
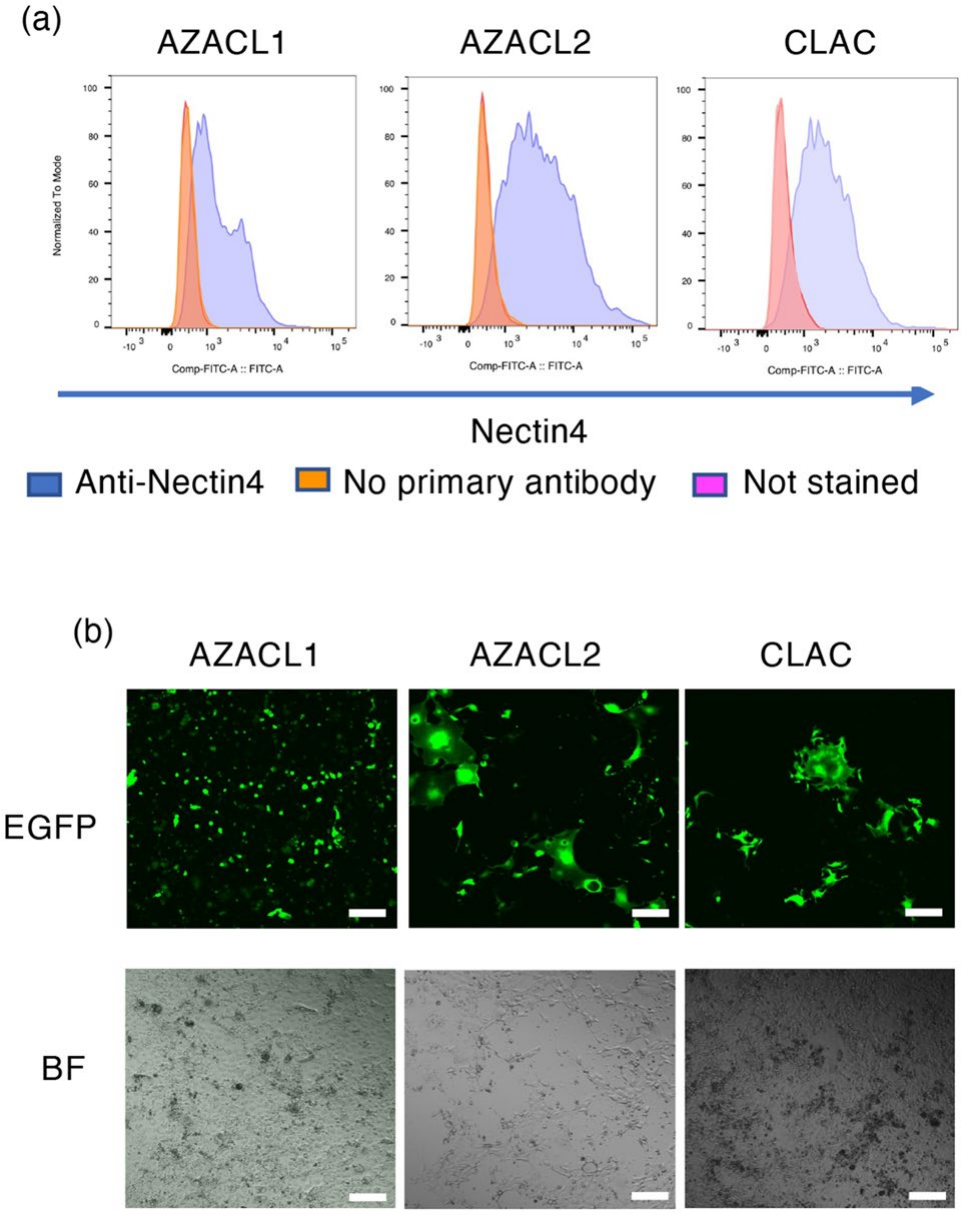
The expression of nectin-4 and the infection of rMV-SLAMblind in canine lung cancer cell lines in vitro. (**a**) The histogram shows the cell surface expression of nectin-4 on AZACL1, AZACL2 and CLAC cells. Purple, stained with anti-nectin-4 antibody; orange, stained without anti-nectin-4 antibody; pink, not stained. (**b**) Fluorescence microscopy of canine lung cancer cells infected with rMV-EGFP-SLAMblind (MOI of 1) at 7 dpi. Corresponding light microscopy photographs (BF; bright field). Scale bar = 10 μm, magnification, 4 × objective lens.

We first evaluated the expression level of nectin-4 in three canine lung cancer cell lines, AZACL1, AZACL2 and CLAC, using flow cytometry. Nectin-4 expression was observed in all three cell lines (Fig. 1a). The mean fluorescence intensity (MFI) values normalized by the value of no primary antibody of cells were the highest in CLAC (1.38 in AZACL1, 1.77 in AZACL2 and 1.83 in CLAC cells).

To evaluate whether rMV-SLAMblind infects the canine lung cancer cells, AZACL1, AZACL2 and CLAC cells were inoculated with rMV-EGFP-SLAMblind, which expresses EGFP, at a multiplicity of infection (MOI) of 1. EGFP signals were detected in AZACL1, AZACL2 and CLAC cells at 7 days post-infection (dpi) (Fig. 1b). This result indicates that rMV-SLAMblind can successfully infect the canine lung cancer cell lines.

**Figure 2:**
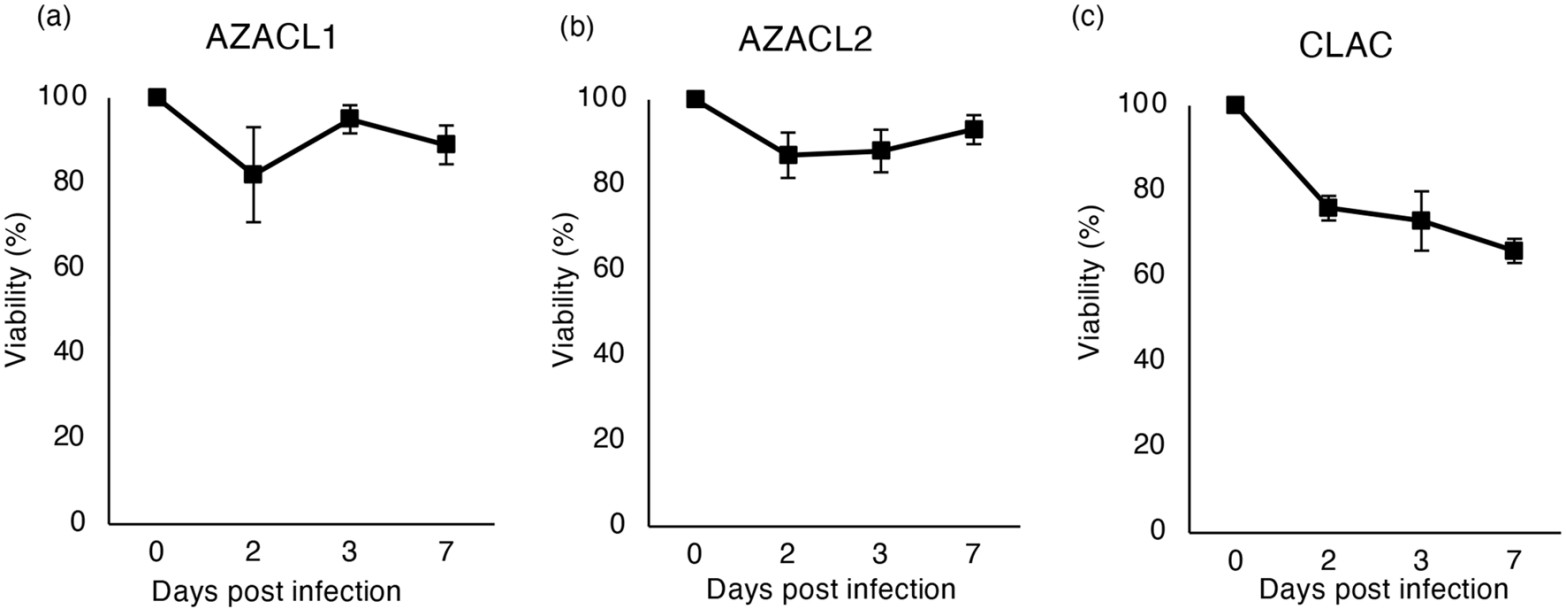
The cell viability of canine lung cancer cell lines infected with rMV-SLAMblind. The cell viability of (**a**) AZACL1, (**b**) AZACL2 and (**c**) CLAC cells after rMV-SLAMblind infection. Cells were infected with rMV-SLAMblind at MOI of 1. Cell viability was measured at 2, 3 and 7 dpi by WST-1 assay. Data are presented as mean ± SD of three independent experiments.

To evaluate the viability of canine lung cancer cells after infection with rMV-SLAMblind, AZACL1, AZACL2 and CLAC cells were infected with rMV-EGFP-SLAMblind at a MOI of 1 and the cell viability was measured 2, 3 and 7 dpi. While the cell viability of AZACL1 and AZACL2 cells were not altered until 7 dpi (Fig. 2a,b), the cell viability of CLAC cells was decreased to 76% at 2 dpi and further decreased to 66% by 7 dpi (Fig. 2c). This result indicates that rMV-SLAMblind shows the highest cytotoxicity on CLAC cells among these three canine lung cancer cell lines. Therefore, we used CLAC cells in subsequent in vivo analyses.

**Figure 3:**
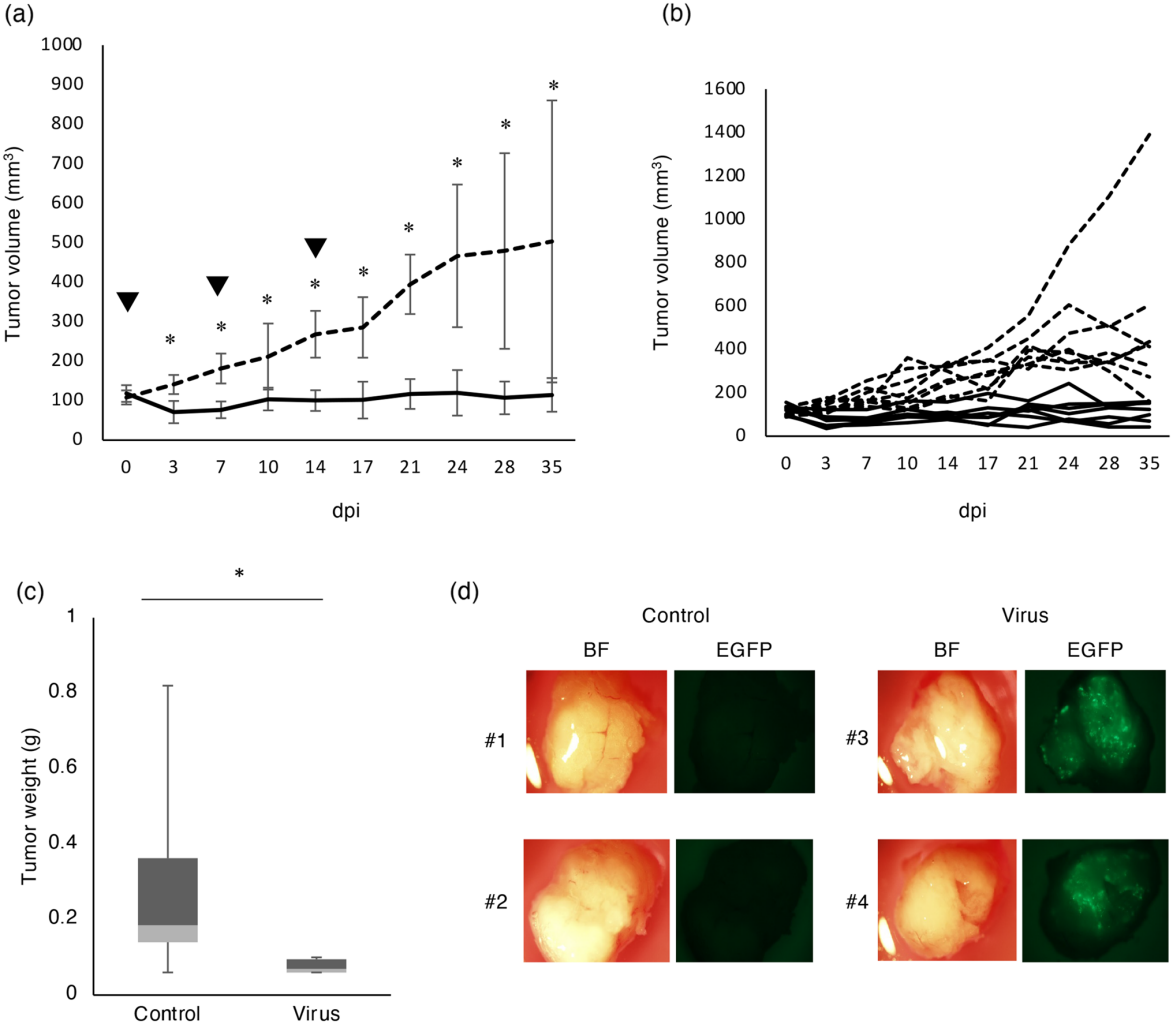
Effects of rMV-EGFP-SLAMblind in the CLAC xenograft model in vivo*.* (**a**) Tumor growth curve of the CLAC xenograft model treated with rMV-EGFP-SLAMblind (n = 7) or saline (n = 8). The arrowhead represents the date of virus administration. Data are shown as mean ± SD. *p < 0.05 using Mann–Whitney *U* test. (**b**) The tumor growth of individual mice. The solid lines and the dotted lines show the tumor volume of the virus treated mice and control mice, respectively. (**c**) Box plot of weight of tumors excised at 35 dpi from the CLAC xenograft models. *p < 0.05 using Welch’s t-test. (**d**) Fluorescence microscopy of excised tumors from the CLAC xenograft models. Corresponding light microscopy photographs. Magnification, 0.63 × objective lens. BF; bright field.

To evaluate the antitumor effect of rMV-SLAMblind on canine lung cancer cells in vivo, CLAC cells were implanted subcutaneously in BALB/cAJcl-nu/nu (nude mice). At 14 days after implantation, 1 × 10^6^ 50% tissue culture infectious dose (TCID_50_) of rMV-EGFP-SLAMblind was intratumorally administered once a week for 3 weeks. The rMV-EGFP-SLAMblind-treated group showed significantly retarded tumor growth compared with the control group (Fig. 3a,b, p < 0.05). At day 35, nude mice were euthanized and tumors were harvested. The tumor weight was significantly decreased in the rMV-EGFP-SLAMblind-treated group in comparison with that of the control group (Fig. 3c, p < 0.05). We confirmed viral growth in tumors from CLAC tumor-bearing mice; EGFP signals were observed in the tumors from the rMV-EGFP-SLAMblind-treated group but not in those from the control group (Fig. 3d). These results suggested that rMV-SLAMblind has an oncolytic effect on the CLAC xenograft model.

### Immune response with rMV-SLAMblind treatment

Virus replication and oncolysis sometimes induce an antitumoral immune response and the release of chemoattractants in the tumor microenvironment^31^. We recently established an immunocompetent mouse model by transplanting human nectin-4-introduced mouse cancer cells and found that intratumoral rMV-SLAMblind treatment activated natural killer (NK) cells and elicited a tumor antigen-specific CD8 T cell response in the tumor microenvironment^32^. Furthermore, rMV-SLAMblind administration resulted in induction of interferon-γ (IFN-γ) in the tumor microenvironment of immunocompetent mouse cancer model. The nude mice used in this study for the xenograft model do not possess mature T cells because of the failure of thymus tissue development, while other immune cells such as NK cells function normally^33^. NK cells are an immune cell subset that exerts cytotoxicity against tumor cells^34^. Therefore, we evaluated the activation of NK cells with rMV-SLAMblind treatment in the CLAC xenograft model.

**Figure 4:**
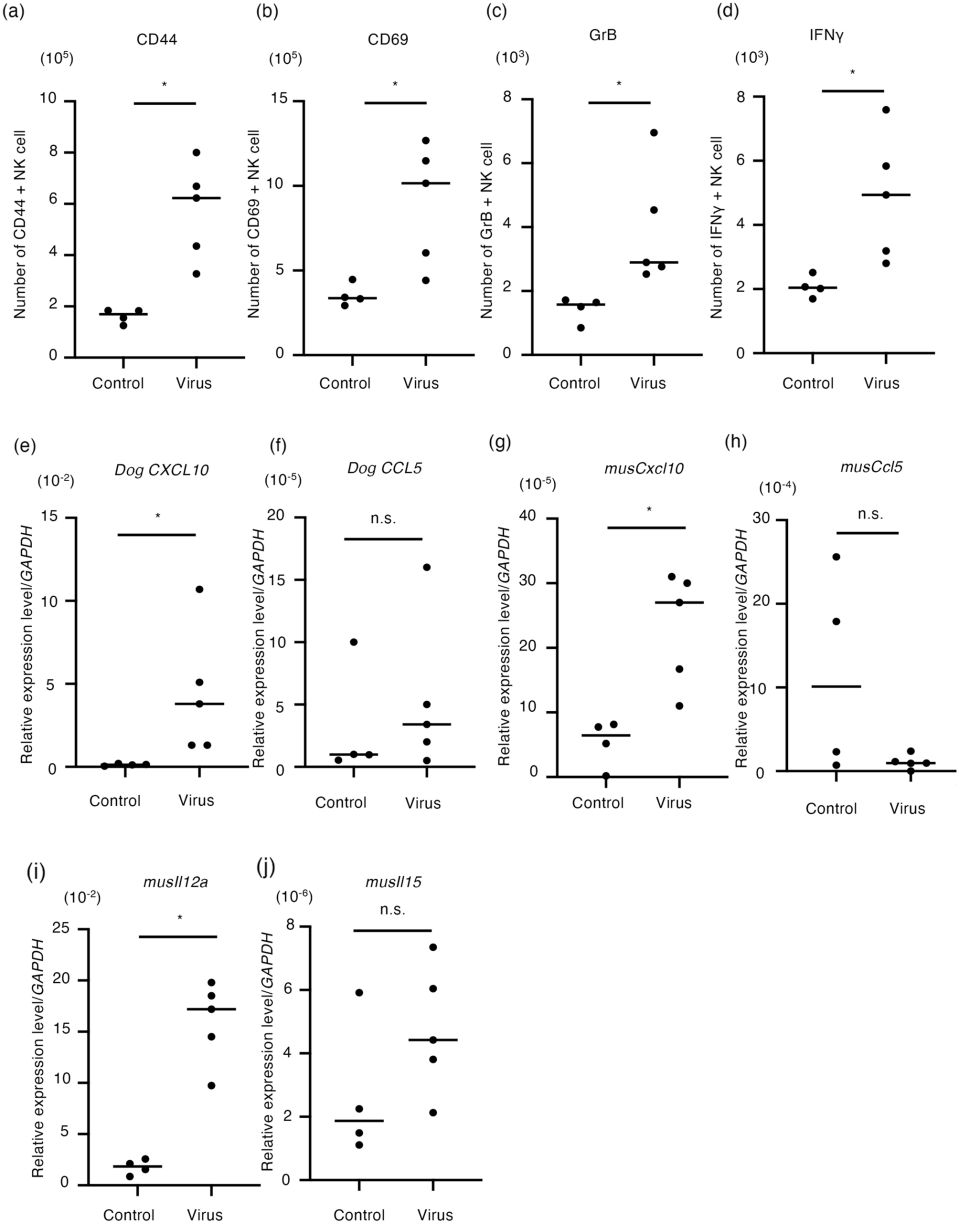
NK cell activation and gene expression related to innate immunity induced by rMV-SLAMblind in the CLAC xenograft model. CLAC tumor-bearing mice were intratumorally administered saline or rMV-SLAMblind. At 3 days after administration, mice were euthanized and collected; lymph node was obtained for FACS analysis and tumor tissue was subjected to gene expression analysis. The number of (**a**) CD 44+ NK cells, (**b**) CD69+ NK cells, (**c**) GrB+ NK cells and (**d**) IFN-γ+ NK cells. Horizontal bars indicate the medians in each CLAC xenograft model. *p < 0.05 using Welch’s t-test. Real time PCR analysis of (**e**) dog *CXCL10*, (**f**) dog *CCL5*, (**g**) mouse *Cxcl10*, (**h**) mouse *Ccl5*, (**i**) mouse *Il12a* and (**j**) mouse *Il15* expression in the tumor tissue of the indicated group. Data were normalized by *Gapdh* from triplicate data. Horizontal bars indicate the medians of relative expression level. *p < 0.05 using Welch’s t-test.

CLAC tumor-bearing nude mice were intratumorally administered with rMV-SLAMblind or saline. At 3 days after administration, mice were euthanized and lymph nodes were collected. The expressions of CD44 and CD69 and those of granzyme B (GrB) and IFN-γ, which is involved in tumor eradication, were analyzed by flow cytometry. The numbers of CD44+ NK cells and CD69+ NK cells were significantly increased in the rMV-SLAMblind-treated group compared with those of the control (Fig. 4a,b, p < 0.05). GrB- and IFN-γ-expressing NK cells were also significantly increased in the rMV-SLAMblind-treated group (Fig. 4c,d, p < 0.05). These results indicate that NK cells were activated in the rMV-SLAMblind-treated CLAC xenograft model.

We then examined the gene expression of cytokines and chemokines in the tumor tissue. The expression levels of dog *CXCL10* and dog *CCL5* in CLAC cells were examined, as a previous report showed that MV infection induced the infected cells to secrete chemokines involved in NK cell recruitment such as CCL5 and CXCL10^35^. While the increase of the expression level of dog *CCL5* was not significant, the expression of dog *CXCL10* was significantly different between rMV-SLAMblind-treated tumor tissue and tumor tissue from the control group (Fig. 4e,f, p < 0.05). Next, to evaluate the activation of non-tumor cells in the tumor tissue in rMV-SLAMblind-injected mice, the gene expressions of mouse *Cxcl10*, mouse *Ccl5*, mouse *Il12a* and mouse *Il15* were examined, because these cytokines that are released from antigen-presenting cells, such as macrophages and DCs, recruit NK cells in tumors (CCL5 and CXCL10) or regulate NK cell activation (IL12a and IL15)^36^. The expression levels of mouse *Cxcl10* and mouse *Il12a* were significantly increased in the tumor tissue of the rMV-SLAMblind treated group (Fig. 4g,i, p < 0.05); however, there was no significant difference in the expression of mouse *Ccl5* and mouse *Il15* compared with the control group (Fig. 4h,j). These results indicated that the rMV-SLAMblind treatment induces the expression of CXCL10 both from tumor cells and non-tumor cells infiltrating in tumor tissue and IL12a from non-tumor cells, which possibly contributes to recruit and activate NK cells.

### Antitumor activity of rMV-SLAMblind in NK cell-depleted mice

**Figure 5:**
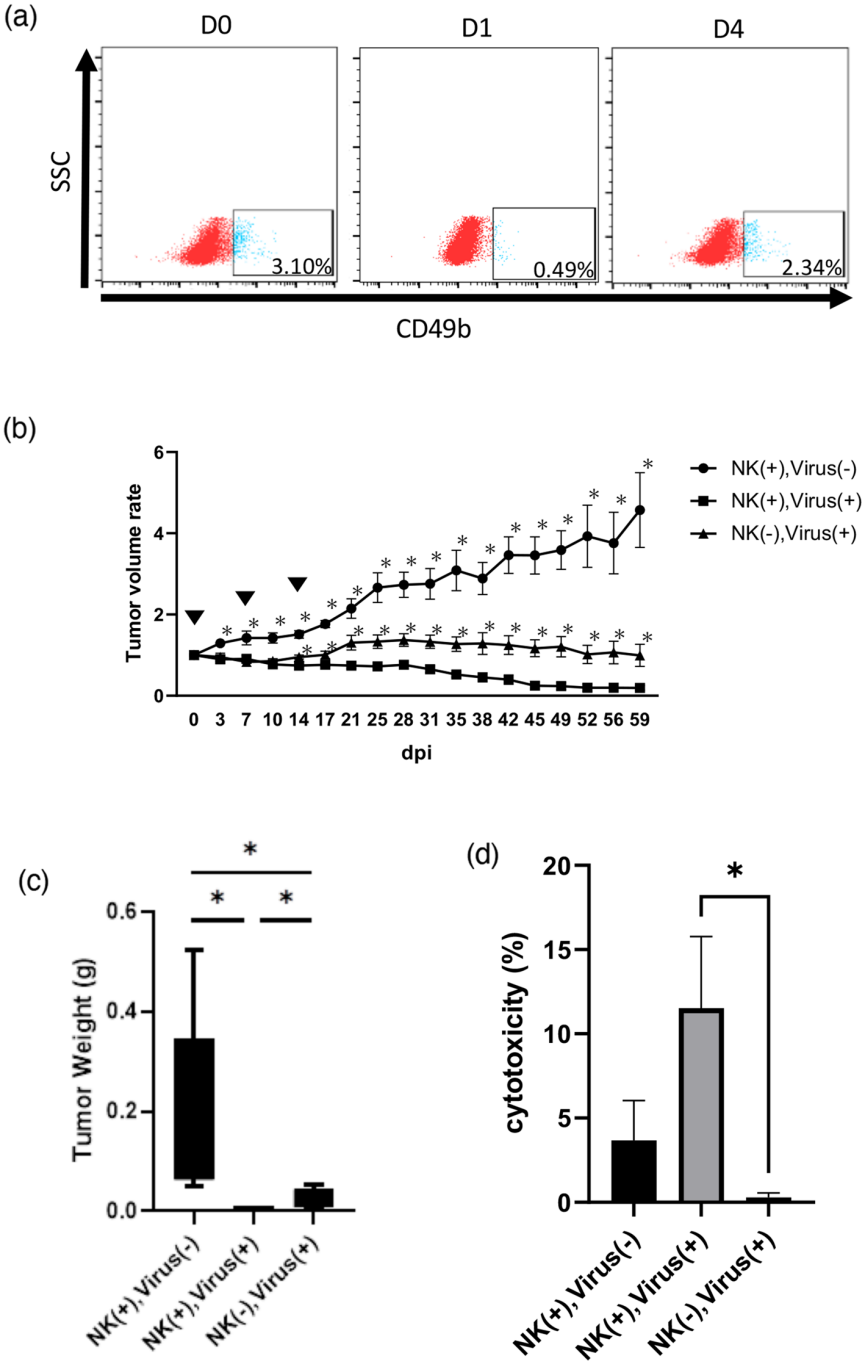
Effects of NK cells on rMV-SLAMblind cancer therapy. Antitumor effects of rMV-SLAMblind in NK-depleted mice were analyzed (n = 6 per group). (**a**) The splenocytes were prepared from mice on day 0, 1, and 4 after administration of anti-asialo GM1 antibody. The ratio of NK cells in splenocytes was measured by staining with anti-CD3 and CD49b antibodies followed by flow cytometry. (**b**) Tumor growth curve treated with rMV-SLAMblind in the mice inoculated anti-asialo GM1 [NK(−), virus (+)], Isotype Control IgG [NK(+), virus (+)], or saline [NK(+), virus (−)]. Tumor volume was shown as relative rate to the volume on day 0. The arrowhead represents the date of virus administration. Data are shown as mean ± SEM. *p < 0.05 using Mann–Whitney *U* test compared to [NK(−), Virus (+)]. (**c**) Box plot of weight of tumors excised at 59 dpi from the xenograft models. *p < 0.05 using Welch’s t-test. (**d**) Tumor cytotoxicity. The splenocytes (5 × 10^5^ cells) were treated with IL-2 (1 ng/ml) for 18 h, then co-cultured with CLAC cells (1 × 10^4^ cells) for 48 h. Cell-free supernatants were analyzed by LDH release assay. *p < 0.05 using Welch’s t-test.

To investigate the role of NK cells in the anti-tumor effect of rMV-SLAMblind, we performed an in vivo cell-specific depletion study. First, depletion of NK cells by anti-asialo GM1 antibody was confirmed. After a single administration, depletion of NK cells was observed after one day, and it was restored after 4 days (Fig. 5a). Hence, the tumor-bearing nude mice (n = 6 per group) were inoculated with antibodies 3 days before and every 3 days after rMV-SLAMblind inoculation. As shown in Fig. 5b,c, the antitumor effect of rMV-SLAMblind treatment was suppressed in NK cell-depleted mice. In addition, the tumor cell-cytotoxicity of NK cell was tested using LDH release as an indicator. Splenocytes were prepared from mice in the above experiment, and then cultured in medium containing IL-2. After 18 h of IL-2 treatment, the splenocytes were cocultured with CLAC target cells for 48 h, followed by the cytotoxicity assay. The results showed that rMV-SLAMblind inoculation increased the cytotoxicity of splenocytes in the nude mice, whereas that was barely observed in NK-depleted mice (Fig. 5d). These results suggest that the antitumor effect of rMV-SLAMblind is enhanced by NK cells.

### Difference in susceptibility to rMV-SLAMblind among canine lung cancer cell lines

**Figure 6:**
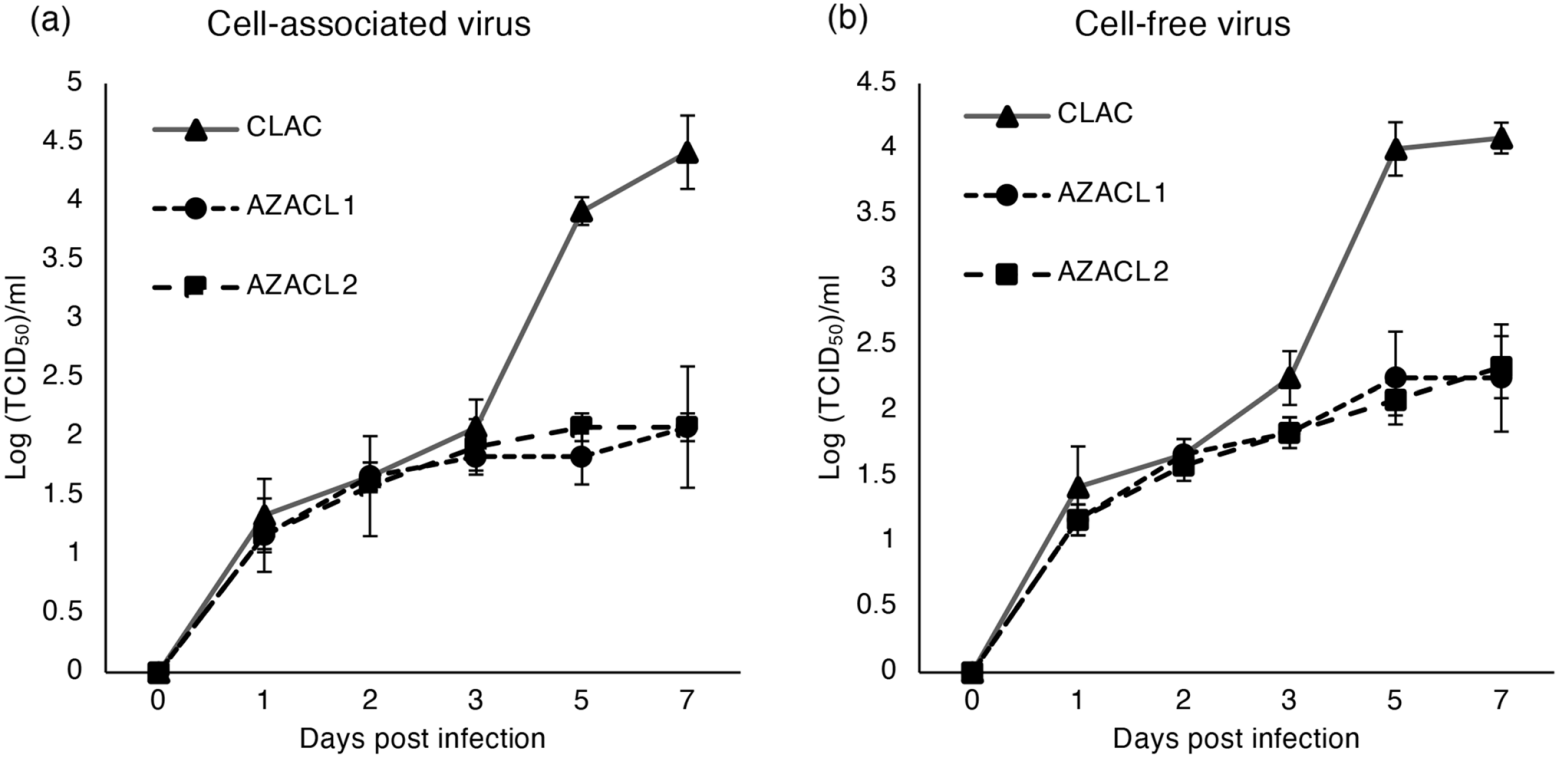
Differential rMV-SLAMblind replication in canine lung cancer cell lines. (**a**,**b**) Viral growth in canine lung cancer cell lines. AZACL1, AZACL2 and CLAC cells were infected with rMV-SLAMblind at MOI of 0.01. The viral titer in (**a**) cell-associated virus and (**b**) cell-free virus were determined at the indicated time points. Data are shown as mean ± SD from triplicate data.

While CLAC cells were susceptible to rMV-SLAMblind, AZACL1 and AZACL2 cells were not efficiently killed by rMV-SLAMblind, in spite of their expression of nectin-4 (Fig. 2). We hypothesized that the growth of rMV-SLAMblind was suppressed in AZACL1 and AZACL2 cells. To compare the replication efficiency of rMV-SLAMblind in the canine lung cancer cell lines, each cell line was infected with rMV-SLAMblind at a MOI of 0.01 and virus growth was examined. The virus titer continued to increase until 7 dpi in CLAC cells, but repressed in AZACL1 and AZACL2 cells, both for cell-associated virus and cell-free virus (Fig. 6a,b). This suggests that the growth of rMV-SLAMblind differs among cell lines and is suppressed in AZACL1 and AZACL2 cells.

**Figure 7:**
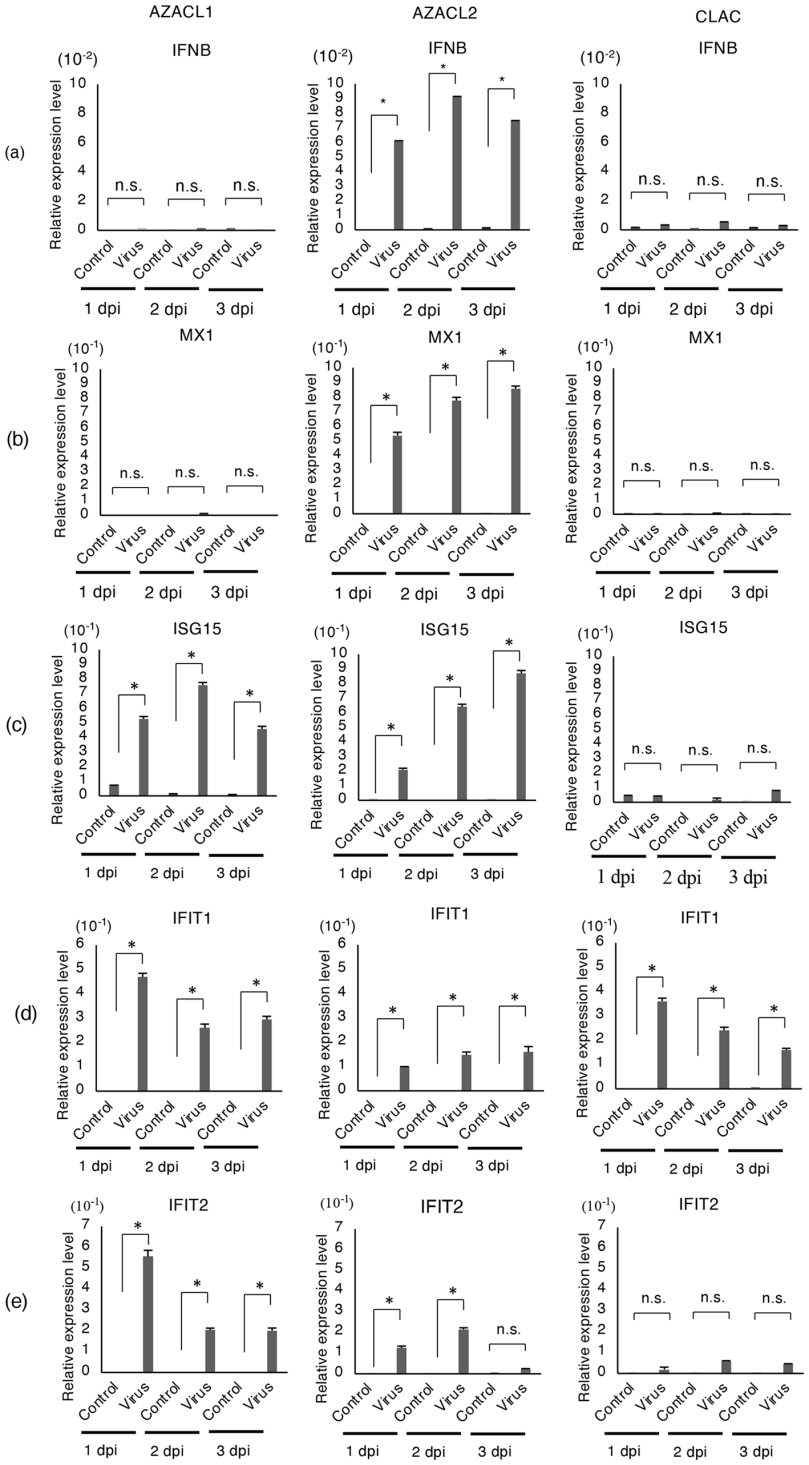
Type 1 IFN and associated gene expression in rMV-SLAMblind infection of canine lung cancer cell lines. (**a**–**e**) AZACL1, AZACL2 and CLAC cells were infected with rMV-SLAMblind at MOI of 1. After 1, 2 and 3 days of incubation, cells were harvested for RNA isolation, followed by real-time PCR. The gene expression of (**a**) *IFNB,* (**b**) *MX1,* (**c**) *ISG15,* (**d**) *IFIT1,* (**e**) *IFIT2* were detected in the canine lung cancer cell lines. The bars indicate the mean value from triplicate data. The error bar represents mean ± SD. Data were normalized by *GAPDH*. *p < 0.05 using Welch’s t-test.

We then hypothesized that the anti-viral response differs among these cell lines. To test this, we evaluated the expression of genes related to the inhibition of virus replication. The relative gene expression level of *IFNB* and the genes downstream of IFN-β signaling such as *MX1, ISG15, IFIT1* and *IFIT2* were investigated. Real-time RT-PCR was performed on the canine lung cancer cell lines infected with rMV-SLAMblind at a MOI of 1. The expressions of *IFNB* and *MX1* were significantly increased in AZACL2 cells at all dpi compared with the control group (Fig. 7a,b, p < 0.05), whereas the expression of *IFNB* was not changed in AZACL1 and CLAC cells. *MX1* was upregulated in AZACL2 cells at all dpi (Fig. 7b, p < 0.05). *ISG15* was significantly increased in AZACL1 and AZACL2 cells at all dpi (Fig. 7c, p < 0.05). The expression of *IFIT1*, which encodes a protein that recognizes the virus, was significantly increased in all cell lines at all dpi (Fig. 7d, p < 0.05). The expression of *IFIT2* was significantly increased in AZACL1 cells at all dpi and in AZACL2 cells at 1 and 2 dpi (Fig. 7e, p < 0.05). These results suggested that the antiviral response differs among these cell lines, and it tends to be suppressed in CLAC cells, a responsive cell line to rMV-SLAMblind.

## Discussion

In this study, we aimed to evaluate the effectivity of rMV-SLAMblind as a tool for canine lung cancer virotherapy. The xenograft model bearing tumors derived from CLAC cells showed a decrease of tumor volume in response to the administration of rMV-SLAMblind as compared with the control group (Fig. 3a), suggesting that rMV-SLAMblind has an antitumor effect on a part of nectin-4-positive canine lung cancer cell lines. In control group, some of the mice showed tumor regression. This may be partly due to the different growth rates and microenvironment in the transplanted CLAC cells.

Oncolytic viruses are genetically modified to infect and amplify preferentially in tumor cells^37^. Some oncolytic viruses lead the cell death not only by infection but also by induction of an anticancer immune response^38^. In this study, we examined the immune response induced by rMV-SLAMblind therapy in tumor cells derived from dog and in non-tumor cells derived from mouse. rMV-SLAMblind therapy significantly increased the expression both of dog *CXCL10* and mouse *Cxcl10* in the tumors (Fig. 4e,g). We also observed the increased expression of mouse *Il12a* (Fig. 4i). These results indicate that rMV-SLAMblind therapy affects not only tumor cells but also non-tumor cells in the tumor microenvironment. *Cxcl10* and *Il12* are expressed in activated DCs in the tumor microenvironment and encode factors that mediate the recruitment and activation of NK cells^39–41^. In fact, we observed significant increase of IFN-γ producing NK cells in the tumor microenvironment following administration of rMV-SLAMblind (Fig. 4d). NK cells mediate apoptosis and perforin/granzyme-mediated cytotoxicity to tumor cells to inhibit tumor growth^42^. Actually, the depletion experiment showed the significant role of NK cells in the anti-tumor efficacy of rMV-SLAMblind (Fig. 5). By using immunocompetent mouse model of melanoma and NK depletion experiment, we recently indicated that rMV-SLAMblind activated NK cells and the activated NK cells contributed to the therapeutic effects^32^. Therefore, rMV-SLAMblind therapy may commonly activate NK cells, contributing its anti-tumor effects.

The killing effect of rMV-SLAMblind in canine lung cancer cell lines correlated with viral growth in the target cells. Infection in CLAC cells, in which rMV-SLAMblind showed the highest effect, generated efficient viral growth, whereas infection in AZACL1 and AZACL2 cells resulted in lower virus growth (Fig. 6a,b). The expression level of nectin-4 was lowest in AZACL1 cells and similar in AZACL2 and CLAC cells (Fig. 1a). This could be one of the reasons why AZACL1 had poor viral growth (Figs. 1a, 6a,b). We evaluated the differences in the gene expression pattern among the three canine lung cancer cell lines after rMV-SLAMblind infection. A study using a vaccine strain of MV showed that basal reduction in the functions of the type 1 IFN pathway is a major contributor to the oncolytic efficacy of MV^43^. In this study, induction of *IFNB* expression was not observed in AZACL1 and CLAC cells (Fig. 7a). This result was not surprising because defects of type1 IFN pathway are common in tumor cells^44,45^. Thus, AZACL1 and CLAC cells possibly have defects in some molecules in this pathway. On the other hand, *IFNB* was up-regulated in AZACL2 cells by rMV-SLAMblind infection (Fig. 7). These results suggested that AZACL2 retains an intact type 1 IFN pathway. In AZACL1, *ISG15* was significantly upregulated, while *IFNB* was not increased (Fig. 7). One possible reason for this is that other molecules such as interferon regulatory factor (IRF) 3 and IFNα may have induced *ISG15*^46^. The IFN signaling and interferon stimulated genes play a pivotal role in cellular defense upon viral infection. Therefore, a difference in the anti-viral state possibly resulted in the difference in resistance against rMV-SLAMblind. Identification of the factors suppressing rMV-SLAMblind replication will be useful to improve virotherapy in future.

In the veterinary field, various viral species have been proposed as oncolytic agents for dogs in recent years^47^. However, limited clinical research on oncolytic virotherapy has been reported, such as zika virus for brain tumors in dogs^48^ and adenovirus in canine melanoma patients^49^. Here we demonstrated that rMV-SLAMblind has antitumor effects on canine lung cancer cells besides mammary tumors^29^ and transitional cell carcinoma^30^. Because the number of cell lines tested in this study was small due to availability of canine lung cancer cell lines, we found only one third of the nectin-4 positive canine cancer cells was killed by rMV-SLAMblind treatment. In the case of human lung cancer cell lines, 6 out of 8 nectin-4 positive cell lines were killed by rMV-SLAMblind treatment^25^. It is reported that human transformed cells showed suppression of induction of *IFNB, MX1*, and *IFIT2* after MV replication^43^. This trend is similar to our results obtained in canine lung cancer cell lines. This implies that basic responses after rMV-SLAMblind infection to cancer cells are conserved between human and dog. On the other hand, as humans are the only natural host of MV, it is possible that there may be difference in replication ability of rMV-SLAMblind and in responses other than IFN response in canine cancer cells.

Future studies including clinical trials of rMV-SLAMblind treatment for cancer in dogs will contribute to generate novel treatments and may be informative for human therapy.

## Methods

### Cell lines and cell culture

AZACL1 and AZACL2 were purchased from Cosmo Bio (Tokyo, Japan) and CLAC (JCRB1453) was obtained from the Japanese Collection of Research Bioresources (Osaka, Japan). These cell lines were derived from canine lung cancer. AZACL1 and AZACL2 cells were maintained in Dulbecco’s Modified Eagle Medium (DMEM) supplemented with 5% fetal calf serum (FCS), 0.295% tryptose phosphate broth, 50 U/ml penicillin and 50 μg/ml streptomycin. CLAC cells were maintained in DMEM supplemented with 5% FCS, 1% non-essential amino acid, 50 U/ml penicillin and 50 μg/ml streptomycin. MCF7 human breast cancer cells (obtained from the Cell Resource Center for the Biomedical Research Institute of Development, Aging and Cancer, Tohoku University, Miyagi, Japan) and Vero/Nectin4 cells we previously established^29^ were maintained in DMEM supplemented with 5% FCS, 50 U/ml penicillin and 50 μg/ml streptomycin. All cells were cultured at 37 °C with 5% CO_2_.

### Virus preparation and titration

rMV-SLAMblind or rMV-EGFP-SLAMblind was propagated and purified as described previously^12^. In brief, MCF7 cells were inoculated with the virus. To release virus particles, the supernatant and infected cells were frozen and thawed three times, followed by sonication and centrifugation at 3000 rpm for 10 min. Viral particles were concentrated by ultracentrifugation at 19,000 rpm for 2 h at 4 °C on a Beckman SW19 rotor (Beckman Coulter, Inc., Brea, CA, USA). The pellet was re-suspended with medium and stored at − 80 °C. Virus titers were determined as TCID_50_/ml by Reed–Muench method using MCF7 cells^50^.

### Virus infection of canine lung cancer cell lines

Canine lung cancer cells (1 × 10^5^) were inoculated with rMV-EGFP-SLAMblind at a MOI of 1. EGFP signal was observed at 7 dpi using a fluorescence microscope (BZ-X710: Keyence, Osaka, Japan).

### Cell viability analysis

Cells (2.5 × 10^4^) were inoculated with rMV-EGFP-SLAMblind at a MOI of 1 and cultured at 37 °C. At 2, 3 and 7 dpi, cell viability was determined using the WST-1 cell proliferation kit (Takara Bio Inc., Shiga, Japan) according to the manufacturer’s protocol. The viability of infected cells was expressed as a percentage by comparison of the noninfected cells.

### Viral growth kinetics

Cells (1 × 10^5^) were infected with rMV-EGFP-SLAMblind at MOI of 0.01 in 12-well plates and incubated in 2% FCS-containing media. We collected cell-free virus (supernatant) and cell-associated virus at 1, 2, 3, 5 and 7 dpi. Cell-associated virus was harvested with three freeze–thaw cycles. The viral titers were determined with Vero/Nectin4 cells.

### Xenograft model

BALB/cAJcl-nu/nu (nude mice, 7-week-old females) were purchased from CLEA Japan (Tokyo, Japan). CLAC cells (1 × 10^6^) were resuspended in 50 μl phosphate buffered saline (PBS) and mixed with an equal volume of Matrigel (BD Matrigel Matrix Growth Factor Reduced; BD Biosciences, San Jose, CA, USA) and 100 μl of the suspension was injected subcutaneously into mice. At 14 days after tumor implantation, mice were randomly divided into two groups and administered 10^6^ TCID_50_ of rMV-EGFP-SLAMblind or saline (Otsuka Pharmaceutical, Tokyo, Japan) intratumorally once a week for three weeks (n = 7 for the virus treated group, n = 8 for control group). The tumor size was measured every 3–4 days, and tumor volume was calculated as (width^2^ × length)/2. At 35 days after virus/saline administration, mice were euthanized under isoflurane anesthesia. The tumor weight was measured and the EGFP signal of the tumor tissue was observed using a fluorescence stereo microscope (MVX10; Olympus).

In the experiments to examine the effects of NK cells, tumor transplantation was performed as described above. When their tumor size grew about 100 mm^3^, mice were randomly divided into three groups and administered 1 × 10^6^ TCID_50_ of rMV-SLAMblind or saline (Otsuka Pharmaceutical, Tokyo, Japan) intratumorally once a week for three weeks (n = 6/group).

### NK cell depletion

NK cell depletion was accomplished via anti-asialo GM1. Briefly, BALB/cAJcl-nu/nu received 100 μg of anti-asialo GM1 antibody (FUJIFILM Wako, Osaka, Japan) or Rabbit IgG Isotype Control (Invitogen) intraperitoneally three days prior to the first infection of rMV-SLAMblind, and every three days thereafter. To confirm NK cell depletion, splenocytes were evaluated by flowcytometry using anti-CD3-PE mAb (145-2C11) and anti-CD49b-FITC mAb (DX5).

### Analysis of immune response

Female nude mice (7-week-old) were inoculated with 1 × 10^6^ of CLAC cells. At 49 days after tumor implantation, the mice were intratumorally administrated 10^6^ TCID_50_ of rMV-EGFP-SLAMblind (n = 5) or saline (n = 4). At 3 days after viral inoculation, mice were euthanized, and the axillary and inguinal lymph nodes of the tumor-injected side were excised. The lymph nodes were passed through a 70 μm cell strainer to obtain single cell suspensions. Live cells were counted by trypan blue exclusion. The single cell suspensions were subjected to flow cytometry analysis.

### Flow cytometry

Cells (5 × 10^5^) were washed in PBS supplemented with 2% FCS (FACS buffer) and incubated on ice for 45 min with goat anti-human nectin-4 polyclonal antibody (R&D Systems, Minneapolis, MN, USA). Cells were washed in FACS buffer and incubated on ice for 45 min with Alexa 488-conjugated anti-goat IgG (Life Technologies, Carlsbad, CA, USA). For immune cell analysis, cells were evaluated using CD44-PE (IM7), CD69-APC (H1.2F3), CD3-Biotin or -PE (145-2C11), CD49b-FITC (DX5), Granzyme B-APC (NGZB), IFN-γ-PECY7 (XMG1.2), Streptavidin-PECy7, TruStain FcX™, Fixable viability dye 506 and control IgG (purchased from BD Biosciences, BioLegend (San Diego, CA, USA) or eBioscience (San Diego, CA, USA)).

To analyze intracellular cytokines, cells were stimulated with 50 ng/ml phorbol-12-myristate13-acetate (PMA; Wako, Osaka, Japan) and 500 ng/ml ionomycin (Wako) in the presence of brefeldin A (eBioscience) for 4 h and then stained with FVD506, followed by surface marker staining. Cell fixation and permeabilization was performed using IC fixation buffer and permeabilization buffer (eBioscience) according to the manufacturer’s instruction; intracellular staining was then conducted. Flow cytometry was performed using a FACS verse flow cytometer (BD Biosciences). Data were analyzed with the Flow Jo software Ver. 9.7.5 (TreeStar, San Carlos, CA, USA).

### Tumor-specific cytotoxicity assay

Spleens were isolated from virus-inoculated and non-inoculated mice, and splenocytes were prepared for cytotoxicity assay. The splenocytes (5 × 10^5^ cells) were treated with IL-2 (1 ng/ml) for 18 h, and followed by co-cultivation with CLAC cells (1 × 10^4^ cells). After 48 h of co-culture, cytotoxicity assay was performed by using LDH-Glo Cytotoxicity Assay kit (Promega, Cat#J2381). The cytotoxicity was calculated as described in the manufacturer’s protocol.

### Analysis of gene expression level

Total RNA was extracted from canine lung cancer cell lines or dissected tumors using TRIzol LS Reagent (Invitrogen, Waltham, MA, USA), followed by DNase treatment by deoxyribonuclease (RT grade; Nippon Gene, Tokyo, Japan) according to the manufacturer’s instruction. PrimeScript™ II 1st strand cDNA Synthesis Kit and oligo (dT) primer was used for cDNA synthesis. To investigate the expression of cytokines in the dissected tumors, the following primers were used: mouse *Il12a* (5′-CGT GAC CAT CAA CAG GGT GA-3′ and 5′-GAG GTA GCT GTG CCA CCT TT-3′), mouse *Ccl5* (5′-CAG CAG CAA GTG CTC CAA TCT T-3′ and 5′-TTC TTG AAC CCA CTT CTC TGG-3′)^51^, mouse *Cxcl10* (5′-GCA ACT GCA TCC ATA TCG ATG AC-3′ and 5′-TGT GCG TGG CTT CAC TCC A-3′)^52^, mouse *Il15* (5′-GTG ACT TTC ATC CCA GTT GC-3′ and 5′-TTC CTT GCA GCC AGA TTC TG-3′)^53^, dog *CXCL10* (5′-CGC TGT ACC TGT ATC AAG ATT AGT G-3′ and 5′-TTG CTT TCA CTA AAC TCT TGA TGG-3′), dog *CCL5* (5′-CTG CTG CTT TGC CTA CAT TTC-3′ and 5′-ACT CAT CTC CAA AGA GTT GAT GTA-3′), dog *IFNB* (5′-AAG CAG CAG TTT GGA GT-3′ and 5′-TGT CCT TCT GGA ACT GG-3′), dog *MX1* (5′-GGA GGC TCT GTC AGG AGT TG-3′ and 5′-TTT GCC TCT CCA CTC ATC CT-3′), dog *ISG15* (5′-AGC AGC AGA TAG CCC TGA AA-3′ and 5′-CAG TTC ACC ACC AGC AG-3′), dog *IFIT1* (5′-GAA GTT TGC AGC TCC CTC CT-3′ and 5′-TGC ATA CCC GGT GCT GAA TT-3′), dog *IFIT2* (5′-GCC AAA CAA TGC CTA CCT GC-3′ and 5′-AGG CGA GAT AGG AGC AGA CA-3′). For the internal control, the following primers were used: *GAPDH* (5′-GCA CCA ACT GCT TAG C-3′ and 5′-TGG ATG CAG GGA TGA TGT TC-3′)^54^; the primers for *GAPDH* were used for both mouse and dog.

For gene expression analysis of dog *CXCL10*, dog *CCL5*, mouse *Cxcl10*, mouse *Ccl5*, mouse *Il12a*, and mouse *Il15* (Fig. 4), the serial dilution of the control plasmid DNA containing the target sequence of each gene was used to construct standard curve. For gene expression analysis of dog *IFNB*, dog *MX1*, dog *ISG15*, dog *IFIT1*, and dog *IFIT2* (Fig. 7), the 2^−ΔΔCT^ method^55^ was used as a relative quantification compared to the control group.

Real-time PCR reaction was performed using Thunderbird Syber qPCR mix (Toyobo, Osaka, Japan) with the Lightcycler 96 system (Roche, Basel, Switzerland). The real-time PCR conditions for amplifying cDNA were as follows: 95 °C for 10 min; 45 cycles of 95 °C for 10 s, 55–60 °C for 30 s and 72 °C for 10 s; and 95 °C for 10 s, 65 °C for 60 s and 97 °C for 1 s.

### Statistical analysis

Statistical analysis was performed with Mann–Whitney *U* test or Welch’s t-test. *p* < 0.05 was considered statistically significant. All the statistical analyses were performed using the GraphPad Prism 8.0 software package (GraphPad, Inc., San Diego, CA, USA).

### Ethical approval

Animal experiments were approved by the Experimental Animal Committee of The University of Tokyo and were performed in accordance with the Regulations for Animal Care and Use of The University of Tokyo. This study was conducted in compliance with the ARRIVE guidelines.

## Data Availability

All data generated or analyzed during this study are included in this manuscript.

## References

[1] Siegel R, Ma J, Zou Z, Jemal A (2014). Cancer statistics, 2014. CA Cancer J. Clin..

[2] Goldstraw P (2016). The IASLC lung cancer staging projects: Proposals for the revision of the TNM stage groupings in the forthcoming (Eighth) edition of the TNM classification of malignant tumors. J. Thorac. Oncol..

[3] Araujo LH, Niederhuber JE (2020). Cancer of the lung: Non-small cell lung cancer and small cell lung cancer. Abeloff’s Clinical oncology.

[4] Griffey SM, Kraegel SA, Madewell BR (1998). Rapid detection of K-ras gene mutations in canine lung cancer using single-strand conformational polymorphism analysis. Carcinogenesis.

[5] Rebhun BR, Culp WTN, Withrow SW (2013). Pulmonary neoplasia. Small Animal Clinical Oncology.

[6] McNiel EA (1984). Evaluation of prognostic factors for dogs with primary lung tumors: 67 cases. J. Am. Vet. Med. Assoc..

[7] Polton G (2018). Survival analysis of dogs with advanced primary lung carcinoma treated by metronomic cyclophosphamide, piroxicam and thalidomide. Vet. Comp. Oncol..

[8] Ogilvie GK (1991). Efficacy of mitoxantrone against various neoplasms in dogs. J. Am. Vet. Med. Assoc..

[9] Tazawa H (2020). Bone and soft tissue sarcoma: A new target for telomerase-specific oncolytic virotherapy. Cancer.

[10] Frohlich A (2020). Talimogene laherparepvec treatment to overcome loco-regional acquired resistance to immune checkpoint blockade in tumor stage3B-4 M1c melanoma patients. Cancer Immunol. Immunother..

[11] Noonan AM (2016). Randomized phase 2 trial of the oncolytic virus pelareorep (Reolysin) in upfront treatment of metastatic pancreatic adenocarcinoma. Mol. Ther..

[12] Sugiyama T (2013). Measles virus selectively blind to signaling lymphocyte activation molecule as a novel oncolytic virus for breast cancer treatment. Gene Ther..

[13] Tatsuo H, Ono N, Tanaka K, Yanagi Y (2000). SLAM (CDw150) is a cellular receptor for measles virus. Nature.

[14] Reymond N (2001). Nectin4/PRR4, a new afadin-associated member of the nectin family that trans-interacts with nectin1/PRR1 through V domain interaction. J. Biol. Chem..

[15] Hao RT (2019). NECTIN4 promotes papillary thyroid cancer cell proliferation, migration, and invasion and triggers EMT by activating AKT. Cancer Manag. Res..

[16] Derycke MS (2010). Nectin4 overexpression in ovarian cancer tissues and serum: Potential role as a serum biomarker. Am. J. Clin. Pathol..

[17] Takano A (2009). Identification of nectin-4 oncoprotein as a diagnostic and therapeutic target for lung cancer. Cancer Res..

[18] Zhang J (2019). Upregulation of nectin4 is associated with ITGB1 and vasculogenic mimicry and may serve as a predictor of poor prognosis in colorectal cancer. Oncol. Lett..

[19] Nishiwada S (2015). Nectin4 expression contributes to tumor proliferation, angiogenesis and patient prognosis in human pancreatic cancer. J. Exp. Clin. Cancer Res..

[20] Takahashi S (2020). A phase I study of enfortumab vedotin in Japanese patients with locally advanced or metastatic urothelial carcinoma. Investig. New Drugs..

[21] Deng H, Shi H, Chen L, Zhou Y, Jiang J (2019). Over expression of Nectin-4 promotes progression of esophageal cancer and correlates with poor prognosis of the patients. Cancer Cell Int..

[22] Zhang Y (2018). High expression of nectin4 is associated with unfavorable prognosis in gastric cancer. Oncol. Lett..

[23] Ma J (2016). Expression and clinical significance of Nectin-4 in hepatocellular carcinoma. Onco Targets Ther..

[24] Rajc J, Gugic D, Frohlich I, Marjanović K, Dumenčić B (2017). Prognostic role of Nectin-4 expression in luminal B (HER2 negative) breast cancer. Pathol. Res. Pract..

[25] Fujiyuki T (2015). A measles virus selectively blind to signaling lymphocytic activation molecule shows anti-tumor activity against lung cancer cells. Oncotarget.

[26] Awano M (2016). Measles virus selectively blind to signaling lymphocyte activity molecule has oncolytic efficacy against nectin-4-expressing pancreatic cancer cell. Cancer Sci..

[27] Fujiyuki T (2020). Recombinant SLAMblind meaales virus is a promising candidate for nectin-4-positive triple negative breast cancer therapy. Mol. Ther. Oncolytics.

[28] Amagai Y (2016). Oncolytic activity of a recombinant measles virus, blind to signaling lymphocyte activation molecule, against colorectal cancer cells. Sci. Rep..

[29] Shoji K (2016). Development of new therapy for canine mammary cancer with recombinant measles virus. Mol. Ther. Oncolytics.

[30] Iizuka K (2020). Antitumor activity of an oncolytic measles virus against canine urinary bladder transitional cell carcinoma cells. Res. Vet. Sci..

[31] Ehrig K (2013). Growth inhibition of different human colorectal cancer xenografts after a single intravenous injection of oncolytic vaccina virus GLV-1h68. J. Transl. Med..

[32] Moritoh K (2023). Immune response elicited in the tumor microenvironment upon rMV-SLAMblind cancer virotherapy. Cancer Sci..

[33] Fogh J, Giovanella BC (1978). The nude mouse. Experimental and Clinical Research.

[34] Adair RA (2013). Cytotoxic and immune mediated killing of human colorectal cancer by reovirus loaded blood and liver mononuclear cells. Int. J. Cancer.

[35] Patterson CE, Daley JK, Echols LA, Lane TE, Rall GF (2003). Measles virus infection induces chemokine synthesis by neurons. J. Immunol..

[36] Bhat R, Rommelaere J (2015). Emerging role of natural killer cells in oncolytic virotherapy. Immunotargets Ther..

[37] Russel SJ, Peng KW (2007). Viruses as anticancer drugs. Trends Pharmacol. Sci..

[38] Russel SJ, Peng KW, Bell JC (2014). Oncolytic virotherapy. Nat. Biotechnol..

[39] Benencia F (2005). HSV oncolytic therapy upregulates interferon-inducible chemokines and recruits immune effector cells in ovarian cancer. Mol. Ther..

[40] Piersma S, Pak-wittel MA, Lin A, Plougastel-Douglas B, Yokoyama WM (2019). Activation receptor-dependent IFN-γ production by NK cells is controlled by transcription, translation, and the proteasome. J. Immunol..

[41] Trinchieri G (2003). Interleukin-12 and the regulation of innate resistance and adaptive immunity. Nat. Rev. Immunol..

[42] Langers I, Renoux VM, Thiry M, Delvenne P, Jacobs N (2012). Natural killer cells: Role in local tumor growth and metastasis. Biologics.

[43] Aref S (2020). Type 1 interferon responses underlie tumor-selective replication of oncolytic measles virus. Mol. Ther..

[44] Stojdl DF, Lichty B, Knowles S, Marius R, Atkins H, Sonenberg N, Bell JC (2000). Exploiting tumor-specific defects in the interferon pathway with a previously unknown oncolytic virus. Nat. Med..

[45] Naik S, Russell SJ (2009). Engineering oncolytic viruses to exploit tumor specific defects in innate immune signaling pathways. Expert Opin. Biol. Ther..

[46] Schoggins JW, Rice CM (2011). Interferon-stimulated genes and their antiviral effector functions. Curr. Opin. Virol..

[47] Sanchez D, Ceserman-maus G, Amandor-molina A, Lizano M (2018). Oncolytic viruses for canine cancer treatment. Cancer.

[48] Kaid C (2020). Safety, tumor reduction, and clinical impact of zika virus injection in dogs with advanced stage brain tumors. Mol. Ther..

[49] Westberg S (2013). Treatment efficacy and immune stimulation by AdCD40L gene therapy of spontaneous canine malignant melanoma. J. Immunother..

[50] Reed LJ, Muench H (1938). A simple method of estimating fifty per cent endpoints. Am. J. Hyg..

[51] Mitsuhashi A (2013). Surfactant protein A suppresses lung cancer progression by regulating the polarization of tumor-associated macrophages. Am. J. Pathol..

[52] Riemer C (2008). Accelerated prion replication in, but prolonged survival times of, prion-infected CXCR3^−^^/^^−^ mice. J. Virol..

[53] Cui G (2014). Characterization of the IL-15 niche in primary and secondary lymphoid organs *in vivo*. Proc. Natl. Acad. Sci. U.S.A..

[54] Tofighi R (2008). Galanin decreases proliferation of PC12 cells and induces apoptosis via its subtype 2 receptor (GalR2). Proc. Natl. Acad. Sci. U.S.A..

[55] Livak KJ, Schmittgen TD (2001). Analysis of relative gene expression data using real-time quantitative PCR and the 2−ΔΔCT method. Methods (San Diego, California).

